# Deviations from the expected relationship between serum FGF23 and other markers in children with CKD: a cross-sectional study

**DOI:** 10.1186/s12882-017-0623-5

**Published:** 2017-06-28

**Authors:** Daisy Liu, Ana Catalina Alvarez-Elías, Brooke Wile, Vladimir Belostotsky, Guido Filler

**Affiliations:** 1 Department of Pediatrics, McMaster University, Hamilton, ON N6A 5W9 Canada; 2 Department of Pediatrics, Schulich School of Medicine & Dentistry, London, ON N6A 5W9 Canada; 3 Department of Pathology and Laboratory Medicine, Schulich School of Medicine & Dentistry, University of Western Ontario, London, ON N5A 5A5 Canada; 4 Department of Medicine, Schulich School of Medicine & Dentistry, University of Western Ontario, 5A 5A5, London, ON Canada; 5 Universidad Nacional Autónoma de México, 04510 Mexico City, Mexico; 6 Laboratorio de Investigación en Nefrología, Hospital Infantil de México Federico Gómez, 067210 Mexico City, Mexico; 7 Department of Pediatrics, Children’s Hospital, London Health Sciences Centre, University of Western Ontario, 800 Commissioners Road East, London, ON N6A 5W9 Canada

**Keywords:** Chronic kidney disease, Fibroblast growth factor 23, pH, Cystatin C (CysC), GFR, Parathyroid hormone (PTH), Vitamin D metabolites

## Abstract

**Background:**

High levels of fibroblast growth factor-23 (FGF23) are associated with mortality. In chronic kidney disease (CKD), FGF23 levels rise as renal function declines. We analyzed the contribution of laboratory values to the variance of FGF23 levels in relationship to a curve of expected FGF23 levels for a given GFR.

**Methods:**

Following approval by the research ethics boards, we measured FGF23, CysC eGFR, creatinine, urea, albumin, calcium, phosphate, vitamin D metabolites, PTH, alkaline phosphatase, CRP, and venous gases in 141 pediatric CKD patients (45, 37, 32, 13 and 14 CKD stages I, II, III, IV, and V, respectively). Data were expressed as median (25th, 75th percentile).

**Results:**

FGF23 correlated significantly with CysC, CysC eGFR, PTH, 1.25 (OH)_2_ vitamin D, phosphate, and pH. The correlation of the latter three remained significant in the multivariate analysis. We calculated a formula for the expected FGF23 value for a given level of eGFR which reads Y = 1295 * e-0.07247*X + 38.35. Deviation by more than 20% from the curve also depended on phosphate, 1.25 (OH)_2_ vitamin D and pH.

**Conclusions:**

Our data emphasize the importance of phosphate and 1.25 (OH)_2_ vitamin D levels. The impact of acidosis on FGF23 warrants further studies.

## Background

Fibroblast growth factor 23 (FGF23) is a 26 kD phosphaturic hormone with a mean level of 35 pg/mL (8.8, 120) in healthy children. [1] It exponentially rises with worsening kidney function and is strongly associated with cardiovascular morbidity [2–7] and mortality in patients with chronic kidney disease (CKD). [8, 9] FGF23 strongly correlates with glomerular filtration rate (GFR) [10, 11] and cystatin C (CysC) in particular. [12] Both FGF23 and CysC are low molecular weight proteins (LMWPs) that accumulate with worsening GFR. It is not yet clear whether higher FGF23 levels occur as a: (i) compensatory response to decreased renal capacity to excrete phosphate [13–15]; (ii) result of the use of calcium-based phosphate binders [16]; (iii) result of decreased 1,25 vitamin D3 levels; (iv) result of secondary hyperparathyroidism [17]; (v) or whether this is related to the disease process of CKD alone, as LMWP blood concentrations are greatly influenced by the GFR. [12, 18, 19] Furthermore, nutritional factors and uremic toxins may also increase FGF23-concentrations. [20, 21] The objective of the current manuscript was to determine expected FGF23 levels for given levels of CysC GFR and to determine which other parameters would be associated with a deviation in FGF23 concentrations.

## Methods

### Study population

We received ethics approval for this cross-sectional study from the University of Western Ontario (REB#16962E) and from McMaster University (REB#12–537). Written informed consent was signed by parents in every case, and in case of a consenting minor, written assent was signed. All patients between the ages of 2 and 18 years with CKD stage I-V at the London Health Sciences Centre, London, Ontario, or McMaster Children’s Hospital, Hamilton, Ontario, were eligible to participate. We considered CKD patients, including those with hyperoxaluria after combined liver-kidney transplantation, cystinosis, ADPKD (albeit these patients have recently been shown to have higher FGF23 levels [22]) or diabetic nephropathy, but excluded those with Jansen’s Disease and autosomal dominant hypophosphatemic rickets as these disorders can affect FGF23 levels. Renal transplant patients were included, but dialysis patients and patients with x-ray-diagnosed vascular calcifications were not considered for this study. Nine patients with tubulopathies and CKD were included even though they may have had biochemical findings such as phosphate and bicarbonate irregularities that could have affected FGF23 levels. This was deemed acceptable as patients with advanced CKD typically have a degree of tubular dysfunction. 193 patients were screened; 49 either did not consent, did not have sufficient samples drawn, or were >18 years of age (the study was originally designed to include young adults up to the age of 40). We recruited 144 pediatric patients without evidence of cardiovascular disease at the various stages of CKD: 40% (I), 21% (II), 21% (III), 10% (IV), and 8% (V). Written and informed consent were obtained for each patient. We also assessed whether the patients received an angiotensin converting enzyme (ACE) inhibitor or an angiotensin II receptor blocker (ARB), calcium carbonate, an erythropoietin stimulating agent (ESA), or an active vitamin D analog. If applicable, a low phosphate diet was prescribed, but we did not monitor adherence. Adjustments were not made for the type of metabolic bone disease, but based on alkaline phosphatase and parathyroid hormone (PTH), none of the patients had adynamic bone disease.

### Laboratory testing

We collected serum to measure FGF23 and CysC levels in conjunction with routine blood work regularly obtained when monitoring pediatric CKD patients. Since CysC concentrations measured before the introduction of the international reference intervals [23] with the Dade Behring assay measure lower, we multiplied CysC measurements by 1.1 in order to ensure that they conform with the new international standards [Conversion factor provided by Dr. Carola Wagner, Siemens Healthcare, personal communication]. Additional measurements included phosphate, calcium, ionized calcium, serum albumin and total protein, bicarbonate, vitamin D metabolites (1, 25-dihydroxy- and 25-hydroxyvitamin D), and intact PTH levels. All were measured using standard laboratory tests. CysC estimated GFR (eGFR) was calculated using the Filler-formula. [24] Serum PTH concentrations were assayed using a solid-phase, two-site chemiluminescent enzyme-labelled immunometric assay (Immunlite 2000 Intact PTH from Diagnostic Products Corporation, Los Angeles, CA, USA).

### Analytical validation of FGF23 assay

Serum FGF23 levels were measured using a sandwich enzyme-linked immunosorbent assay (ELISA) system that incorporates two types of monoclonal antibodies, requiring the presence of both the N-terminal and C-terminal portions of FGF23 (Kainos Laboratories, Inc., Tokyo, Japan; Millipore, St. Charles, Missouri, USA). The manufacturers’ instructions were followed and each antibody-coated well contained 50 μL of serum sample and 50 μl of assay diluent. Over a span of 2 h the plate was then incubated at room temperature on a plate mixer. After 4 washes, the plate was incubated with FGF23 conjugate, mixing for 1 h at room temperature. Substrate was then added and allowed to develop for 30 min following another 4 washes. The signal was read in a microplate reader at 450 nm absorbance within 10mins. [25] Inter-assay and intra-assay coefficients of variation were 5.0 and 3.0%, respectively. CysC was measured using the Siemens Healthcare nepholometric assay (PETIA) on a BN-Prospec platform (Dade-Behring). [24, 26]

### Data analysis

Simple descriptive statistics were used whenever possible. Contiguous data were subject to normality testing using the Shapiro-Wilk normality test. Normally distributed data was analyzed using parametric methods (mean, standard deviation, t-test, Pearson correlation, ANOVA), while the other data were analyzed using nonparametric methods (median, 25th percentile, 75th percentile, Mann Whitney t-test and Spearman rank correlation). We classified correlations based on the rho value as follows: strong correlation >0.70, medium correlation 0.30–0.60, mild correlation <0.30. Non-linear curve fitting (one-phase exponential decay) generated the function of the non-linear regression model for FGF23 and eGFR, formulating a line of expected FGF23 concentrations with their corresponding eGFR levels. We chose one-phase exponential associations because the small molecular weight of FGF23 should behave similarly to that of CysC. Contingency tables were analyzed using the Chi square test. We then calculated the percentage above and below the expected concentration for each FGF23 value, classifying each as within, below, or above 20% of the expected value. Twenty percent was chosen so to have roughly equal numbers of measurements within the expected range as well as above and below the expected range. This analysis was limited to higher stages of CKD as only negligible deviation occurred in lower stages of CKD. Finally, we compared all parameters with regard to these three categories to identify which laboratory measures explained a level outside 20% of the expected value using ANOVA. The last calculations were limited to patients with CKD stage III-V. No adjustments were made for missing data. All statistical analyses were performed using the statistical software GraphPad Prism version 5.0 (GraphPad Inc., San Diego, CA, USA) and IBM SPSS Statistics version 21 (IBM Canada Corporation, Markham, ON, Canada). A *p*-value of <0.05 was considered statistically significant.

## Results

Patient characteristics are given in Table 1. Forty-one patients had congenital anomalies of the kidney and urinary tract (CAKUT), 34 were renal transplant recipients, 28 had a glomerulopathy or glomerulonephritis (mostly focal and segmental glomerulosclerosis), 11 had hemolytic uremic syndrome (HUS), nine patients had a tubulopathy, and 17 had other diagnoses. CKD stage variation was as follows: CKD stage I (eGFR > 90 mL/min/1.73 m^2^): 40%; CKD stage II (eGFR 60–89 mL/min/1.73 m^2^); 21%, CKD stage III (eGFR 30–59 mL/min/1.73 m^2^); 21%, CKD stage IV (eGFR 15–29 mL/min/1.73 m^2^); 10% and CKD stage V (eGFR <15 mL/min/1.73 m^2^): 8%. Three patients with CKD stage II received bicarbonate. Of the 51 patients with CKD stage III-V, 23 received ACE or ARB therapy, 16 received calcium carbonate, 1 received amphojel, 1 received renagel, 24 received bicarbonate, 6 received potassium citrate, 18 received ESA, and 19 received vitamin D analogs. Only 5 patients received recombinant human growth hormone.

**Table 1:** Clinical and laboratory parameters of the 144 patients with CKD stages I-V

Parameter	Unit	Mean or Median, as appropriate	STD or IQR, as appropriate
Age	years	12	(6.5, 16.0)
Gender			
Height	cm	140.7	(116.6, 160.1)
Weight	kg	36.6	(20.8, 53.4)
Systolic Blood pressure	mm Hg	108	13
Diastolic Blood Pressure	mm Hg	65	11
pH		7.36	0.05
CO_2_	mmol/L	48	(41, 52)
Bicarbonate	mmol/L	26	(23, 28)
Urea	mmol/L	7.4	(5.0, 13.4)
Creat	umol/L	74	(51, 155)
Ht/Creat Ratio	cm * L/umol	2.09	(1.38, 3.3)
CysC	mg/L	1.20	(0.88 2.16)
CysC eGFR	mL/min/1.73 m^2^	74	(39, 106)
CysCxPTH	mg x pmol/L^2^	4267	(2687, 6620)
FGF23	pg/mL	50	(37.17, 90.16)
Calcium	mmol/L	2.37	(2.27, 2.47)
Phosphate	mmol/L	1.31	(1.19, 1.49)
25-OH Vit D	nmol/L	64	(49.95, 87.05)
1,25 (OH)_2_ Vit D	pmol/L	76	(50.5, 109.5)
Alk Phos	U/L	184	(102.5, 246)
PTH	pmol/L	5.9	(3.65, 11.3)
Ionized Ca	mmol/L	1.16	(1.1, 1.2)
CRP	mg/L	1.05	(0.6, 3.5)
Albumin	g/L	43	(39.25, 46)
Total Protein	g/L	71	(61, 74)
U_Ca_	mmol/L	0.74	(0.46, 1.985)
U_Creat_	mmol/L	5	(2.8, 9.2)
U_Alb_	mg/L	31.9	(7.8, 121.5)
MA/Cr Ratio	mg /mmol	8.7	(1.9, 37.3)

**Fig. 1:**
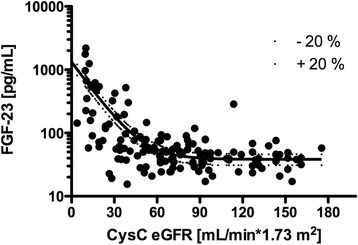
Non-linear regression analysis of FGF23 as a function of Cystatin C eGFR. *Lines* representing 20% above and 20% below the expected concentration are also shown. This curve can be described as Y = Span * e^-K*X^ + Plateau, where the half-life of the decay is 0.6932/K, Y is the expected FGF23 concentration, X is the eGFR and the curve begins at Span + Plateau and ends at Plateau. The values of the curve were: Span = 1295, K = 0.07247, Plateau = 38.35 and Halflife = 9.565

Since previous studies have shown that CysC moderately correlates with FGF23 concentrations, we calculated a predicted curve of expected FGF23 levels (ΔFGF23) for given CysC eGFR values (Fig. 1). We then performed a correlation analysis of the key laboratory measures, both for the FGF23 concentrations and the deviation from the predicted line for a given level of GFR (Table 2). Phosphate correlated strongly with FGF23, and PTH correlated moderately and positively with FGF23. While 1,25(OH)_2_ vitamin D levels correlated mildly and negatively with FGF23, 25(OH) vitamin D did not. There was no adjustment for 1,25(OH)_2_ vitamin D analogue supplementation. As expected, CysC eGFR correlated moderately and negatively with FGF23.

**Table 2:** Correlations between parameters that have interaction with FGF23

*n* = 144	CysC eGFR [mL/min/1.73 m^2^]	Phosphate [mmol/L]	Ionized Calcium	1,25 (OH)2 Vit D [pmol/L]	PTH [pmol/L]	pH	Bicarbonate	CRP
FGF23 [pg/mL] (r-rho, *p*)	−0.548 0.000^*^	0.667 0.000^*^	−0.360 0.000^*^	−0.338 0.000^*^	0.516 0.000^*^	0.213 0.029^*^	0.156 0.071	0.243 0.004^*^
ΔFGF23 [pg/mL] (r-rho, *p*)	0.193 0.021^*^	0.329 0.000^*^	−0.144 0.107	−0.043 0.628	0.205 0.018^*^	0.219 0.026^*^	0.029 0.743	0.048 0.585

*denotes significant associations (*p* < 0.05)

*FGF23* Fibroblast Growth Factor 23, *CysC* Cystatin C, *Vit* Vitamin, *PTH* Parathyroid Hormone

ΔFGF23 was calculated as measured FGF23 - expected FGF23

As seen in Fig. 1, the expected FGF23 for a given cystatin C concentration can be calculated as Y = 1295 ^*^ e^-0.07247*X^ + 38.35, where X is a given cystatin C concentration and Y is the expected FGF23 concentration

**Fig. 2:**
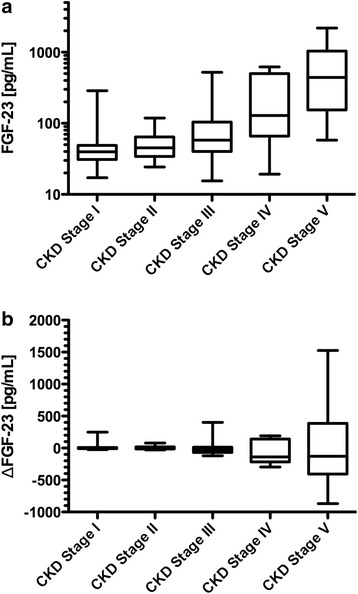
This box-and-whisker plot expresses the raises in FGF23 as eGFR decreases, with a significant *p*-value (*p* < 0.0001, ANOVA), as expected. Data are depicted as median (mid line of the box), 25th (bottom line of the box), and 75th percentile (top line of the box) and minimum and maximum (end of the whiskers). ΔFGF23 between the expected and measured values was not significant, but variability worsened with worsening CKD stages

We analyzed FGF23 by CKD stage and the deviation from the expected FGF23 level for a given eGFR using box-plot graphs (Fig. 2). While FGF23 minimally deviated from the expected values in CKD stages I and II, CKD stage V was found to have the largest deviation (Fig. 2).

We then used linear regression analysis (even though some of the parameters were not normally distributed) to determine the univariate components of the variance of FGF23. Phosphate correlated moderately (r^2^ = 0.441, *p* = 0.000), and PTH (r^2^ = 0.261, *p* = 0.028) and 1,25(OH)_2_ Vitamin D (r2 = 0.107, *p* = 0.000) correlated weakly with FGF in the univariate linear regressions.

Forty-four percent of overall FGF23 variance may be explained by phosphate, while 26% of elevated FGF23 may be explained by PTH and 11% by a decrease in 1,25(OH)_2_ vitamin D levels. Rises in FGF23 were associated with lower levels of calcium and 1,25(OH)_2_ vitamin D levels (r^2^ = 0.123, *p* = 0.000; r^2^ = 0.107, *p* = 0.000). Table 3 lists the differences between markers that interact with FGF23 at the different stages of CKD.

**Table 3:** Differences between parameters that interact with FGF23 in the CKD stages

*n* = 144	CKD Stage I *n* = 59	CKD Stage II *n* = 31	CKD Stage III *n* = 29	CKD Stage IV *n* = 14	CKD Stage V *n* = 11	*p*
Cystatin C eGFR [mL/min/1.73 m^2^] Median (IQR)	126.56 (101.85–159.61)	74.65 (69.43–79.08)	42.30 (36.76–53.67)	21.47 (17.87–26.58)	11.53 (10.69–13.76)	0.000^*^
FGF23 [pg/mL] Median (IQR)	40.45 (31.66–50.00)	45.19 (34.51–55.32)	63.79 (41.27–162.94)	133.64 (75.05–539.91)	535.44 (158.45–1249.95)	0.000^*^
Delta FGF23 [pg/mL] Median (IQR)	1.79 (−8.17 to 11.14)	−6.68 (−15.58 to 7.89)	−25.39 (−81.04 to 14.73)	−144.33 (−276.82 to 82.31)	17.17 (−437.13 to 682.81)	0.111
Phosphate [mmol/L] Median (IQR)	1.26 (1.07–1.38)	1.25 (1.15–1.38)	1.26 (1.19–1.44)	1.62 (1.37–1.88)	2.18 (1.51–3.17)	0.000^*^
Ionized Calcium [mmol/L] Mean SD	1.15 ± 0.06	1.17 ± 0.10	1.16 ± 0.093	1.10 ± 0.070	1.02 ± 0.083	0.000^*^
1,25 (OH)_2_ Vit D [pmol/L] Median (IQR)	89.00 (70.75–128.25)	79.00 (62.00–121.50)	60.00 (36.50–81.00)	45.50 (21.50–89.25)	11.00 (10.00–34.00)	0.000^*^
PTH [pmol/L] Median (IQR)	3.90 (2.80–5.50)	5.10 (3.75–8.20)	9.15 (7.17–12.52)	17.50 (11.52–70.27)	43.65 (7.20–112.65)	0.000^*^
pH Mean SD	7.36 ± 0.041	7.34 ± 0.033	7.34 ± 0.052	7.30 ± 0.080	7.39 ± 0.076	0.003^*^
Bicarbonate [mmol/L] Mean SD	26.39 ± 3.27	24.58 ± 3.24	24.30 ± 3.98	28.08 ± 7.47	30.30 ± 4.44	0.000^*^
CRP Median (IQR)	1.00 (0.59–3.67)	0.59 (0.56–2.82)	1.55 (0.59–3.60)	0.59 (0.59–4.85)	3.55 (2.55–10.90)	0.010^*^

*denotes significant associations (*p* < 0.05)

One-way ANOVA and Kruskal-Wallis test. For a significant *p* < 0.05

To assess the interdependency and collinearity of the parameters, we then performed a multivariate analysis using logistic binary regression. Although we wanted to test all five of the parameters that correlated with FGF23 (phosphate, PTH, 1,25 (OH)_2_ vitamin D, ionized calcium and bicarbonate level), the model became unstable if more than 4 parameters were examined at the same time. We therefore detailed 5 different combinations with four variables for each model to assess the impact of all parameters that correlated with FGF23. We used a cut-off point of >230 pg/mL for FGF23 in all of the models. In the first model, the two most important variables that predicted higher FGF23 levels were CKD stage and phosphate, even when controlling for 1,25(OH)_2_ vitamin D levels and PTH. In Model 2, only 1,25(OH)_2_ vitamin D levels remained significant when controlling for the covariates. Maintaining a 1,25(OH)_2_ vitamin D level > 50 pmol/L was a protective factor for high FGF23 levels. In Model 3, 1,25(OH)_2_ vitamin D levels and phosphate were the only significant parameters after controlling for the covariates. Maintaining a phosphate level < 1.60 mmol/L was protective for maintaining an FGF23 level < 230 pg/mL in 95% of cases. In Model 4, neither CRP nor bicarbonate level influenced FGF23 after controlling for covariates. Finally, in Model 5, a pH >7.35 was associated with higher FGF23 levels, even after controlling for all covariates. The analysis suggests that correcting renal acidosis over a pH of 7.35 may be associated with a significant increase in the risk of elevated FGF23 levels.

**Fig. 3:**
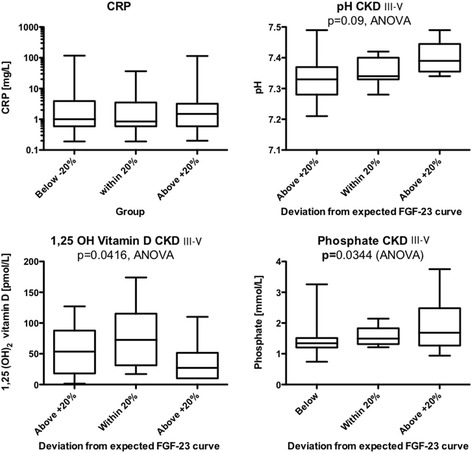
pH and phosphate levels of patients with CKD stages 3–5 in relationship to being within 20% or above or below the expected FGF23 concentration

Finally, we analyzed which parameters accounted for values within 20% of the expected FGF23 level, or below and above the 20% band. Only 1,25(OH)_2_ vitamin D and phosphate differed between the three groups. Interestingly, overcorrected pH tended to have the worst FGF23 levels. Figure 3 selectively shows C-reactive protein, pH, 1,25(OH)_2_ vitamin D and phosphate. A lower phosphate level and a higher 1,25(OH)_2_ vitamin D level were protective. Moreover, we tested whether the use of ACE or ARB, calcium carbonate, ESA, or vitamin D analogs was associated with higher than expected FGF23 levels. Whereas more patients with FGF23 levels below or at the expected range received ACE inhibitors (47.4% versus 38.5%) and about the same proportion received vitamin D analogs (31.6% versus 30.8%), this did not reach statistical significance. In contrast, patients with FGF23 levels below the expected range took about the same proportion of calcium carbonate (31.6% versus 30.8%), and less ESA (36.8% versus 44.4%), although this did not reach statistical significance.

## Discussion

Our results confirmed previous findings showing a moderate, non-linear correlation between FGF23 and CysC. [12] However, using an appropriate multivariate analysis, only the effects of phosphate and 1,25(OH)_2_ vitamin D levels were statistically significant. The correlation between FGF23 and CysC is not surprising, given that both are low molecular weight proteins of similar size that accumulate in CKD. Using a one-phase exponential decay model, we calculated expected FGF23 levels for a given CysC eGFR and analyzed the relationship between the deviation of the expected value from the measured value and various clinical laboratory parameters. Only the effects of 1,25(OH)_2_ vitamin D and phosphate levels remained significant. Lower phosphate levels and higher vitamin D levels were protective, suggesting that normalizing both parameters may form the most important strategy in reducing FGF23 concentrations. Therapy with ACE or ARBs, calcium carbonate, ESAs and active vitamin D was not associated with lower or higher FGF23 levels than expected. Finally, this comprehensive analysis revealed a novel, previously unreported association between pH and FGF23 levels. However, pH in venous blood is not a robust biomarker and at this point in time, the impact of the acid-base state on FGF23 requires further study. The data do stress the importance of serum phosphate and 1,25(OH)_2_ vitamin D levels for FGF23 levels, although the optimal target concentrations remain to be established.

The relationship between FGF23 levels and 1,25(OH)_2_ vitamin D levels is well described. [27] Experimental studies demonstrate that direct administration of recombinant FGF23 induces a dose-dependent decrease in renal CYP27B1 mRNA expression, an increase in renal 24-hydroxylase mRNA expression, and a consequent decrease in serum 1,25(OH)_2_ vitamin D concentrations. [28] Phosphate is also an independent marker of vascular disease in CKD [29] and the importance of lowering this marker has been shown in previous studies. [28] We could not find an association between ACE inhibition (mostly Ramipril, data not shown) and FGF23 levels, in contrast to recent findings in patients with diabetic nephropathy. [30]

The most unexpected finding in our comprehensive study on biomarker associations with FGF23 concentrations was its association with venous pH. This association has not been described in detail and remained significant even after correcting for all covariates. However, we must acknowledge that a venous blood gas was analyzed, which is the weakest means of measuring pH and is potentially heavily influenced by pre-analytical error. However, the venous blood gas was determined in an identical fashion without undue delay during the transport to the lab. Also, pH affects many systems and covariables and the changes in pH may only be associated with FGF-23 increase without inferring causality. Assuming this association between pH and FGF23 deviation exists, we must interpret this finding: We do know that pH affects serum calcium concentrations and normally corrects ionized calcium for a pH of 7.40, so calcium concentrations are higher with a lower pH. It is understandable that FGF23 would be influenced by pH due to variability in calcium, which in turn regulates FGF23 expression in bone. [31] Still, ionized calcium was no longer significant after controlling for covariates (Table 4). It is common practice to correct metabolic acidosis in patients with CKD. [32] Experimental and clinical studies suggest that correcting acidosis using alkali therapy attenuates these complications and improves quality of life, although this has not yet been established. [32] Our results suggest that correcting metabolic acidosis as well as avoiding a pH of >7.35 in the venous blood gas analysis may be important in achieving lower FGF23 concentrations. This recommendation needs to be considered carefully because of the limitations of this study, including the impact of the concomitant calcium carbonate, bicarbonate, and potassium citrate supplementation. The growth hormone/insulin-like growth factor I axis may also be implicated. Unfortunately, our numbers were too small to perform a subgroup analysis.

**Table 4:** Binary Logistic Regression analysis of five models assessing for collinearity as described in the text

MODEL 1. Condition: FGF23 > 230 pg/mL In the presence of: Phosphate, 1,25 (OH)_2_ Vit D, PTH and CKD Stage.			
Parameter	SE	OR (CI 95%)	*p*
Phosphate [mmol/L] Below 1.60	0.948	0.056 (0.009–0.357)	0.002*
1,25 (OH)_2_ Vit D [pmol/L] Over 50	1.033	0.400 (0.053–3.028)	0.375
PTH [pmol/L]	0.016	1.000 (0.969–1.032)	0.968
CKD Stage Cystatin C eGFR [mL/min/1.73 m^2^]	0.483	2.940 (1.141–7.575)	0.026*
MODEL 2. Condition: FGF23 > 230 pg/mL In the presence of: Bicarbonate, 1,25 (OH)_2_ Vit D, PTH and Ionized Calcium.			
Parameter	SE	OR (CI 95%)	*p*
Bicarbonate [mmol/L]	0.107	0.936 (0.760–1.154)	0.536
1,25 (OH)_2_ Vit D [pmol/L] Over 50	0.850	0.138 (0.026–0.728)	0.020*
PTH [pmol/L]	0.014	1.026 (0.998–1.053)	0.066
Ionized Calcium [mmol/L]	4.753	0.002 (0.000–23.520)	0.195
MODEL 3. Condition: FGF23 > 230 pg/mL In the presence of: 1,25 (OH)_2_ Vit D, PTH, Ionized Calcium and Phosphate.			
Parameter	SE	OR (CI 95%)	*p*
1,25 (OH)_2_ Vit D [pmol/L] Over 50	0.975	0.100 (0.015–0.673)	0.018*
PTH [pmol/L]	0.016	1.014 (0.983–1.047)	0.379
Ionized Calcium [mmol/L]	4.431	7.980 (0.001–467.56)	0.641
Phosphate [mmol/L] Below 1.60	0.923	0.045 (0.007–0.277)	0.001^*^
MODEL 4. Condition: FGF23 > 230 pg/mL In the presence of: Bicarbonate, CRP, 1,25 (OH)_2_ Vit D and PTH.			
Parameter	SE	OR (CI 95%)	*p*
Bicarbonate [mmol/L]	0.082	1.021 (0.868–1.200)	0.804
CRP	0.038	0.987 (0.917–1.063)	0.735
1,25 (OH)_2_ Vit D [pmol/L] Over 50	0.788	0.110 (0.024–0.517)	0.005*
PTH [pmol/L]	0.014	1.027 (1.000–1.055)	0.053
MODEL 5. Condition: pH > 7.35 In the presence of: FGF23, Ionized Calcium, Bicarbonate and CKD Stage.			
Parameter	SE	OR (CI 95%)	*p*
FGF23 [pg/mL]	0.005	1.009 (1.000–1.019)	0.047*
Ionized Calcium [mmol/L]	3.325	0.028 (0.000–18.791)	0.281
Bicarbonate [mmol/L]	0.064	0.913 (0.806–1.035)	0.155
CKD Stage Cystatin C eGFR [mL/min/1.73 m^2^]	0.252	0.588 (0.359–0.964)	0.035*

*denotes significant associations (*p* < 0.05)

*SE* Standard error, *OR* Odds ratio, *CI* Confident interval. For a significant *p* value <0.05

Major strengths of our study include the sizeable patient number, the proportion of patients with CKD stage III or greater, and the robust inclusion of multiple covariates, including CysC, a superior marker of GFR. [33] Building onto what is known about the relationship between FGF23 and CysC, this study increases our knowledge of an area that is woefully understudied. [12, 34, 35] The development of a formula (Y = 1295 * e-0.07247*X + 38.35, where Y is the FGF23 concentration in pg/mL and X is the given CysC eGFR level in mL/min/1.73 m^2^) to calculate the expected FGF23 concentration for a given level of eGFR is also novel and may help clinicians better interpret FGF23 levels.

Our study also has several limitations. As this is a cross-sectional study, associations, even when thoroughly checked using binary logistic regressions, do not denote causality. The role of inflammation could also not have been studied comprehensively enough because we did not use a micro-CRP assay and a large number of CRP values had to be set to the lower detection limit, which may have obscured a significant association. Some preliminary data does suggest that FGF23 levels may be lower with lower levels of micro-CRP. [36] Furthermore, some conditions that were included such as ADPKD may be associated with higher FGF23 levels when compared to other causes of CKD. [22] Theoretically FGF23 levels could be higher in patients with cystinosis, but a PubMed search (search string “Cystinosis AND FGF23”) revealed no publications. Only one patient of the patients with tubulopathy had cystinosis with advanced CKD and the FGF23 concentration was not outside of the expected range. Klotho was also not measured. pH is a major outcome variable in our study, but it is only a venous pH and we had no way to control for the possibility of hyperventilation. Arterial or capillary venous blood gas studies are not routinely employed in these patients. A venous blood gas pH of 7.35 likely corresponds to an arterial blood gas pH of 7.40. pH can modify calcium levels, but also the sensitivity of the calcium-receptor and thus PTH metabolism. [37] Although other confounders may be responsible, these were not elucidated in our univariate analysis. Krieger et al. recently showed in a neonatal mouse model that metabolic acidosis induces increased FGF23 in bone. [38] It should be noted that neonatal kidneys differ between mice and humans as nephrogenesis only continues after birth in rodents. Our findings regarding pH must be confirmed in prospective studies before these findings are translated into practice. The study was also underpowered for a multivariate analysis with more than 4 covariates. We did, however, solve this problem by employing multiple models for the binary logistic regression. The study also lacked the capacity to study the patients without vitamin D analogue therapy separately.

## Conclusions

Nonetheless, the creation of a formula that is able to provide the expected FGF23 level for a given level of eGFR is a step towards improving our knowledge and clinical application of FGF23 in CKD. Our comprehensive analysis confirms the importance of phosphate and 1,25 (OH)_2_ vitamin D levels in relation to FGF23. The relationship between FGF23 and pH has not previously been described and warrants further study. The study findings must be confirmed in additional prospective studies such as in the CKiD study.

## Abbreviations

ACE: Angiotensin converting enzyme inhibitor
ARB: Angiotensin II receptor blocker
CAKUT: Congenital anomalies of the kidney and urinary tract
CKD: Chronic kidney disease
CysC: cystatin C
eGFR: estimated GFR
ELISA: Enzyme-linked immunosorbent assay
ESA: Erythropoietin stimulating agent
FGF23: fibroblast growth factor-23
GFR: glomerular filtration rate
HUS: Hemolytic uremic syndrome
LMWPs: Low molecular weight proteins
PTH: Parathyroid hormone
